# Age-related patterns in distal skin temperature during naps

**DOI:** 10.1093/sleep/zsag077

**Published:** 2026-03-17

**Authors:** Severine Soltani, Wendy Hartogensis, Patrick Kasl, Subhasis Dasgupta, Stephan Dilchert, Frederick M Hecht, Benjamin L Smarr, Ashley E Mason

**Affiliations:** Bioinformatics and Systems Biology Graduate Program, University of California San Diego, La Jolla, CA, United States; Shu Chien-Gene Lay Department of Bioengineering, University of California San Diego, La Jolla, CA, United States; Osher Center for Integrative Health, University of California San Francisco, San Francisco, CA, United States; Shu Chien-Gene Lay Department of Bioengineering, University of California San Diego, La Jolla, CA, United States; San Diego Supercomputer Center, University of California San Diego, La Jolla, CA, United States; Zicklin School of Business, Baruch College, The City University of New York, New York, NY, United States; Osher Center for Integrative Health, University of California San Francisco, San Francisco, CA, United States; Shu Chien-Gene Lay Department of Bioengineering, University of California San Diego, La Jolla, CA, United States; Halıcıoğlu Data Science Institute, University of California San Diego, La Jolla, CA, United States; Osher Center for Integrative Health, University of California San Francisco, San Francisco, CA, United States

**Keywords:** aging, biomarkers, daytime sleep, naps, siesta, statistics, sleepiness, sleep pressure, thermoregulation

## Abstract

**Study Objectives:**

Naps are common worldwide; despite their prevalence, physiological changes during naps are less well-characterized than during nighttime sleep. We aimed to characterize napping patterns and their associated physiological changes across age groups.

**Methods:**

We used longitudinal wearable device data from 20 027 individuals from the TemPredict Study to assess (1) napping frequency and timing and (2) distal skin temperature changes around naps.

**Results:**

Older age groups napped more frequently and consistently throughout the week. Individuals aged 30–49 years showed the greatest increase in nap frequency from weekdays to weekends, whereas younger (18–19 years) and older (≥60 years) age groups showed the smallest increases. Sleep timing and distal skin temperature rhythms appeared tightly coupled, even during the daytime. Both sleep timing and distal skin temperature rhythms appeared phase-delayed among younger age groups relative to older age groups. Distal skin temperature was higher during naps on weekends relative to weekdays. Older individuals (≥65 years) had lower distal skin temperatures during naps relative to younger individuals (≤30 years) (weekends: *p* < .0001; *δ* = −0.20; weekdays: *p* < .0001; *δ* = −0.19). Longer naps were generally associated with greater distal skin temperature before napping, especially among younger individuals (≤30 years).

**Conclusion:**

We observed age-related differences in napping patterns and distal skin temperature around and during naps. Future research should examine whether such distal skin temperature changes around naps relate to sleep pressure and age-related disease risk.

## Graphical Abstract

**Graphical Abstract:**
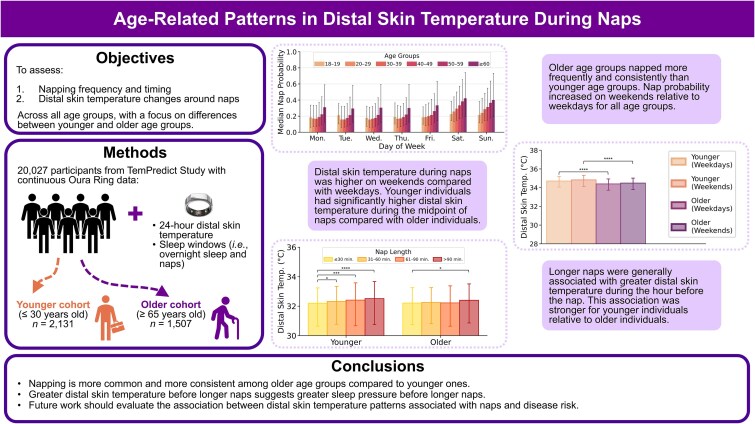

Statement of Significance We present a rich analysis of naps and their associated physiological changes in a real-world setting from a large, heterogeneous population. Building on prior work linking distal skin temperature to sleep–wake regulation, we characterized distal skin temperature patterns across the day, including during naps. We observed that older age groups napped more frequently and consistently. In contrast, younger age groups had higher distal skin temperature during naps. Younger age groups also showed a stronger association between nap duration and distal skin temperature before the nap. These data provide insight into daytime sleep–wake physiology changes across the lifespan. Longitudinal monitoring of distal skin temperature holds promise for assessing daytime sleep physiology and its association with sleep pressure and age-related health outcomes.

## Introduction

Naps are short, daytime bouts of sleep usually associated with the afternoon [1]. Naps are part of the daily routine across cultures globally (e.g. Spanish *siesta* [2], Chinese *wujiau* [3], Italian *riposo* [4], and Arabic *qailulah* [5]), but have become less common in the context of modern-day school, work, and social routines [4]. Given the historical prevalence of naps, it appears likely that naps may have served a biological purpose or were at least a natural behavior [6]. Indeed, naps appear to play an important–yet paradoxical–role both in maintaining health and indicating disease risk [7]. Napping can serve to consolidate memory [8] and promote emotional well-being [9]; however, napping can also reflect an attempt to catch up on “sleep debt” attributable to lacking restorative nighttime sleep [10]. Naps may also be an indicator of sleep–wake cycle dysregulation, which is a common occurrence of aging and implicated in Alzheimer’s disease and related dementias (ADRD) [11]. The increased tendency to nap or take lengthy naps has been implicated in cardiovascular disease [12, 13], diabetes [14, 15], and respiratory illness [16, 17]. Thus, naps can both provide health benefits and indicate disease states.

Modern-day school, work, and social routines, which typically preclude time in the afternoon for napping, impact people’s opportunities to nap. It is unclear at present whether napping (1) is a natural part of human circadian rhythms (and in some contexts, frequently suppressed due to modern-day routines) or (2) represents a deleterious departure from daily patterns of physiological regulation. This differentiation could be important for understanding whether suppressing naps may be harmful. For example, previous work has shown that suppressing nighttime sleep is harmful: shift workers whose work schedules conflict with the natural nighttime sleep–wake cycle (i.e. night and swing shift workers) are more likely to experience adverse health effects than day shift workers [18]. Thus, characterizing physiological changes associated with naps may clarify whether some naps or napping habits may be harmful.

Body temperature regulation is critical for the regulation of sleep–wake cycles. Sleep onset is typically preceded by a decrease in core body temperature, driven by heat loss through the extremities, which results in a rise in distal skin temperature [19]. Most research on distal skin temperature changes associated with sleep focuses mainly on nighttime sleep. Laboratory studies have shown that distal skin temperature increases during naps under controlled conditions [20]. However, little is known about how these temperature dynamics manifest during naps or times when naps tend to occur in everyday settings across the lifespan. Further characterization of changes in skin temperature during and around naps may provide insight into daytime sleep propensity across populations. For example, previous studies have observed distal skin temperature increases at habitual bedtime [21], and that the application of heat to increase distal skin temperature may be useful for shortening sleep onset latency for nighttime sleep [22]. Researchers have yet to uncover the extent to which distal skin temperature may play a role in reflecting as well as influencing sleep propensity in the context of daytime sleep (i.e. naps) in real-world contexts.

Previous work, though limited, has shown that individuals with diseases associated with daytime sleepiness experience greater increases in distal skin temperature across the daytime relative to controls [23–25]. These findings suggest an association between daytime distal skin temperature and sleep–wake dynamics. However, few studies have examined how distal skin temperature changes during naps or times when naps tend to occur, and even fewer have characterized these changes across age groups. Addressing these gaps may provide novel insights into physiological markers of daytime sleep pressure.

In this study, we used longitudinal wearable device data from 20 027 participants from the TemPredict Study [26] to characterize changes in distal skin temperature around naps. We assessed (1) napping frequency and timing (i.e. time of day and day of week) and (2) distal skin temperature changes around naps and times when naps tend to occur. In particular, we focused on characterizing age-related differences in these domains. We hypothesized that because older adults typically nap more often than younger adults [27], and because blood flow to the skin becomes less efficient with age [28], younger and older adults may exhibit different physiological changes in distal skin temperature around naps and times when naps tend to occur.

## Materials and Methods

### Study overview

Mason et al. (2022) outline initial participant recruitment in [26]: Briefly, Mason et al. (2022) recruited participants on a rolling basis for the TemPredict Study from March 19 to September 23, 2020. Participants were eligible to enroll in this study if they were (1) at least 18 years of age, (2) able to communicate in English, and (3) had a smartphone. Recruitment occurred from (1) existing Oura Ring users who received an in-app invitation to join the study and (2) frontline healthcare workers at participating institutions who were provided with Oura Rings and study materials after IRB approval at their sites. Existing Oura Ring users were distributed globally, while frontline healthcare workers were recruited from major U.S. academic medical centers and affiliated hospitals (e.g. University of California San Francisco [UCSF] hospitals at Mission Bay and Parnassus; Stanford Medical Center; Northwestern McGaw Medical Center; Beth Israel Deaconess Medical Center, Harvard Medical School; University of Miami Health System; and University of Texas Southwestern Medical Center Dallas, among others).

Existing Oura ring users were able to share pre-recorded data from the Oura Ring spanning back to January 1, 2020. All participants were able to opt into sharing their data through November 30, 2020, marking the end of data collection and follow-up for the TemPredict Study. All participants provided electronic informed consent before enrollment. UCSF Institutional Review Board (#20-30 408) and the U.S. Department of Defense Human Research Protections Office (#E01877.1A) approved all study activities. All research was performed in accordance with relevant guidelines and regulations and the Declaration of Helsinki. Study methods and results are reported following the Strengthening the Reporting of Observational Studies in Epidemiology Statement for observational studies [29].

All participants wore the Oura Ring Gen2 (Ouraring.com), a commercially available wearable sensor device (Oura Health Oy, Oulu, Finland), on a finger of their choosing. We used participants’ Oura Ring sleep data; the Oura Ring sleep staging algorithm that was applied to this dataset has been validated against polysomnography [30]. The Oura Ring measures distal skin temperature using a negative temperature coefficient thermistor (resolution of 0.07°C) located on the internal surface of the ring. While Oura Ring is designed for continuous use during daily activities, participation in the TemPredict study did not require a minimum duration of Oura Ring wear-time or number of data points.

Participants completed a baseline survey that they received by email from UCSF (Qualtrics [31]); wherein they reported demographics (e.g. sex, age, race, country) and other relevant healthcare information, such as an ongoing pregnancy. Participants completed daily surveys by tapping on a survey link within the Oura App on their personal smartphone that led to a survey hosted by UCSF (Qualtrics). In these daily surveys, participants reported symptoms of illness (e.g. fever, cough, fatigue, and loss of taste/smell) as well as information on COVID-19 and influenza diagnosis dates. Participants completed monthly surveys by clicking on a link sent to them by email from UCSF (Qualtrics); participants were able to report updated healthcare information, such as a new pregnancy in these surveys. Importantly, unlike smartwatches, the Oura Rings were sized to each individual, limiting its usability by multiple individuals.

### Software

We used Python 3.11.9 [32] and the computational resources of the National Research Platform [33]. Supplementary Table S1 outlines all Python packages we used.

### Data preparation and selection

**Figure 1:**
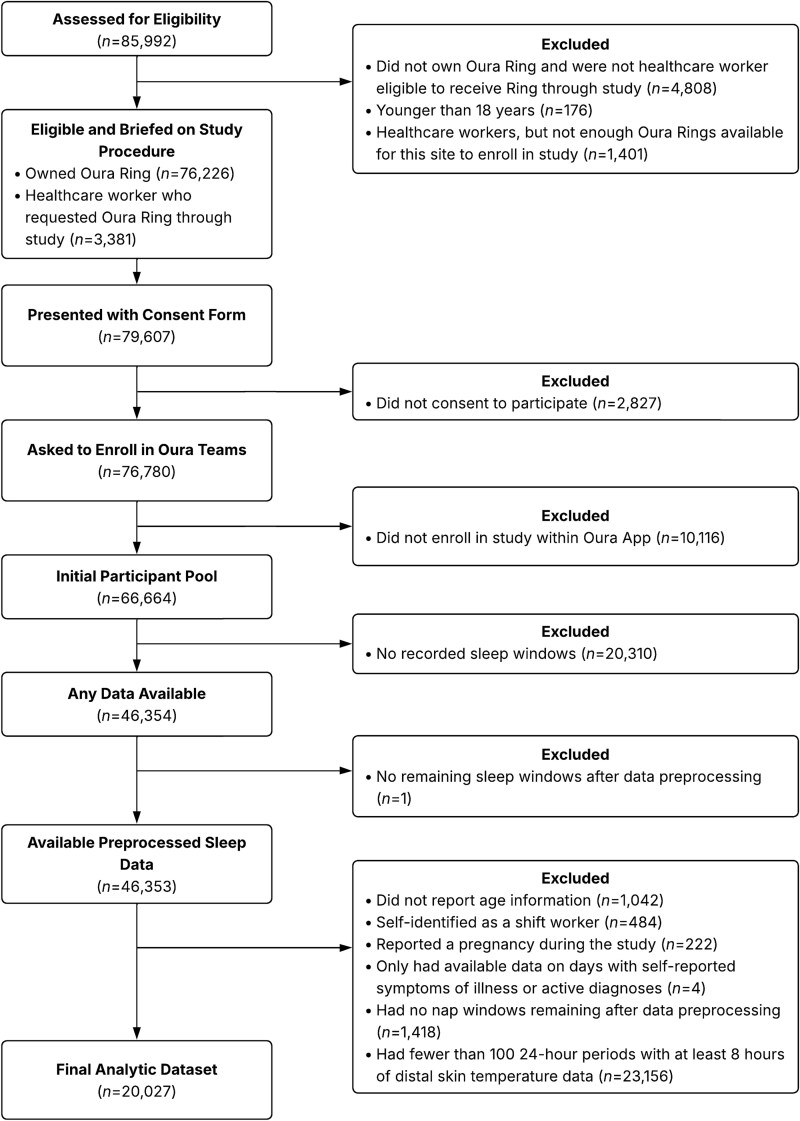
Flow diagram of participants for analytic sample selection.

The original TemPredict Study included an initial participant pool of 65 319 individuals [26]. Since then, 1345 more individuals’ in-app identifiers were able to be matched to their study identifiers. Thus, a subsequent study publication by Mason et al. (2024) included an initial participant pool of 66 664 individuals [34]. We provided a flow diagram of participant inclusion/exclusion criteria for this study in Figure 1, with our initial steps being identical to those of Mason et al. (2024) [34]. Of these 66 664 participants, *n* = 20 310 had sleep window data, as provided by Oura. Oura Ring sleep data contain separate records for each detected sleep window. Supplementary Material section *Definitions, Data Preparation, and Data Selection* provides a detailed definition of what we considered naps and overnight sleep for the purpose of these analyses, and details on the following data preprocessing steps for the remaining 46 354 individuals: We preprocessed the sleep window data by (1) merging sleep windows with other nearby windows to consolidate them into a single, longer window; (2) filtering out short sleep windows of less than 15 min (which excluded one individual for whom this step eliminated all sleep windows), to mitigate against the possibility of the Oura Ring designating periods of inactivity as sleep; and (3) removing naps close to overnight sleep windows, which are more likely to represent early portions of polyphasic overnight sleep or potentially false sleep windows due to the Oura sleep staging algorithm being primed to detect sleep towards the evening. After taking these steps, the longest sleep window in a 24-h period (as identified by the Oura Ring) was defined as the overnight sleep window, and remaining short windows were considered naps. Importantly, if no longest sleep window was identified within a 24-h period, we did not promote the longest short sleep window to overnight sleep. After preprocessing the sleep window data, we excluded individuals who (1) did not report age information (*n* = 1042); (2) self-identified as a shift worker (*n* = 484); (3) reported a pregnancy during the study (*n* = 222); (4) only had data on days where they reported symptoms of illness or active diagnoses (*n* = 4); (5) had fewer than 100 24-h periods in which at least 8 h of skin temperature data were recorded (*n* = 23 156); or (6) had no nap windows remaining follow the data preprocessing steps (*n* = 1418). These steps yield a final analytic sample of 20 027 individuals.

### Variable preparation

#### Daily nap probability

For each participant, we calculated nap probability by dividing the number of recorded naps by the number of days on which the person had at least 2 h of Oura Ring data from ring wear during the daytime hours (i.e. separate from overnight sleep). We defined such days as “daytime wear days” (to distinguish from days when Oura Ring use primarily occurred during overnight sleep) and used 2 h as the threshold for a daytime wear day because most naps are 2 h or shorter. We computed this metric for each participant who had at least one “daytime wear day” on each day of the week (i.e. at least one Monday daytime wear day, at least one Tuesday daytime wear day).

#### Daily sleep probability

We defined daily sleep probability as the probability of being asleep at each minute of each day of each week. We calculated daily sleep probability for each individual by first tallying whether they were asleep (asleep = 1, awake = 0) at each minute of each day across all days of their data. For each minute of the week (i.e. 10 080 total minutes from Monday to Sunday), we calculated the mean across each minute of the individual’s data by summing the binary sleep indicator values and dividing by the number of weeks of available data. This procedure produced an estimate of the probability of being asleep at each minute of each day of the week. We used the mean rather than the median to retain small increases in daytime sleep probability (i.e. naps) that would otherwise be reduced to 0 if computing median sleep probability.

#### Daily distal skin temperature

We calculated daily distal skin temperature for each individual by first aligning their distal skin temperature data from Monday to Sunday by minute of week (i.e. we compared the same minute on the same day of the week, across all weeks with data, which gave us 10 080 potential minutes of data from each week). We generated a daily distal skin temperature profile (e.g. a “Monday profile”) for each individual by computing the median distal skin temperature for each of those 10 080 min of the week, across all weeks.

#### Nap time midpoint (t_midnap_)

We defined *t*_midnap_ for each nap as the timestamp at the midpoint of the nap (i.e. halfway from nap onset to nap completion).

#### Pre-nap time window (t_nap-X-Y_)

We defined *t*_nap*-*X*-*Y_ as the time window from X to Y in minutes before nap onset time, for example, *t*_nap*-*0*-*5_ (0–5 min before nap onset time), *t*_nap*-*5*-*10_ (5–10 min before nap onset time), and *t*_nap*-*0*-*60_ (0–60 min before nap onset time).

#### Nap time window (t_nap + X-Y_)

We defined *t*_nap + X*-*Y_ as the time window from X to Y in minutes after nap onset time, for example, *t*_nap + 10-15_ (10–15 min after nap onset time).

#### Wake time window after overnight sleep (t_wake + X-Y_)

We defined *t*_wake + X-Y_ as the window from X to Y in minutes after an individual’s final awakening time after overnight sleep, for example, *t*_wake+120-180_ (120–180 min after wake time), and *t*_wake+180-240_ (180–240 min after wake time).

### Analyses by outcome

#### Daily nap probability across the week by age group

To assess weekday-weekend differences in daily nap frequency, we used a linear mixed model (LMM) with a random intercept for the individual and fixed effects for age and day of week (i.e. weekends = 1 vs. weekdays = 0).

To visualize these data, we calculated daily nap probability for each age group (i.e. 18–19, 20–29, 30–39, 40–49, 50–59, and ≥60 years) and plotted the median and interquartile range (IQR) of nap probability to determine nap frequency for each age group.

#### Daily sleep probability across the week by age group

To visualize the effects of weekday vs. weekend and age on median sleep probability, we stratified weekly minute-level sleep probability by age group (18–19, 20–29, 30–39, 40–49, 50–59, and ≥60 years). We then plotted daily sleep probability on a logarithmic scale to highlight lower sleep probabilities (smaller values) by age group.

#### Daily distal skin temperature across the week by age group

To determine the effects of weekday vs. weekend and age on distal skin temperature, we used an LMM with a random intercept for the individual and fixed effects for age and day of week (i.e. weekends = 1 vs. weekdays = 0) to predict median daily sleep probability during *t*_midnap_. To visualize these data, we plotted the median daily distal skin temperature profiles for each age group (18–19, 20–29, 30–39, 40–49, 50–59, and ≥60 years). We computed distal skin temperature during naps by computing distal skin temperature at *t*_midnap_.

To assess weekday vs. weekend-related changes in distal skin temperature in different age groups, we limited our analysis to individuals categorized as either “older” (≥65 years) or “younger” (≤30 years). We selected 65 years as the threshold for older individuals because it historically represents the retirement age in the United States and serves as a proxy for reduced weekday workplace obligations; in contrast, adults aged 30 years or less in the United States are typically in school or working. The younger group was selected to represent the opposite end of the adult age spectrum, allowing for a clearer contrast in age-related patterns. For older (*n* = 1507) and younger (*n* = 2131) individuals, we plotted the median distal skin temperature across each minute of the day under two conditions: weekdays with naps and weekends with naps. We then computed the distal skin temperature during the midpoint of naps (*t*_midnap_) stratified by weekdays and weekends. We calculated and plotted the distribution of distal skin temperatures for weekday vs. weekend and distal skin temperature at *t*_midnap_. We compared distal skin temperature distributions of weekend and weekday at *t*_midnap_ using the Mann–Whitney *U* test and reported Cliff’s *δ* as a measure of effect size [35].

#### Nap length and distal skin temperature

To examine how distal skin temperature before naps affects nap duration, for younger (≤30 years) and older individuals (≥65 years), we calculated the median distal skin temperature during a 60-min window before each nap (i.e. *t*_nap-0-60_) for naps lasting ≤30, 31–60, 61–90, or >90 min. We included individuals in this analysis if they had at least one nap of each duration (≤30, 31–60, 61–90, and >90 min). Among younger and older individuals, we used median distal skin temperature during *t*_nap-0-60_ for naps of duration ≤30 min as the reference against which we compared *t*_nap-0-60_ for naps of varying durations using the Mann–Whitney *U* test and Cliff’s *δ*.

#### Distal skin temperature changes before naps

To describe changes in distal skin temperature that occur before nap time, we computed median distal skin temperature for 5-min segments at *t*_nap-0-5_ (5 min prior to nap) and *t*_nap-5-10_ (5–10 min prior to nap) and created a pair of values for each nap. We then calculated median distal skin temperature across all segments to obtain one pair of values per individual for each *t*_nap-0-5_ and *t*_nap-5-10_.

We regressed median distal skin temperature during *t*_nap-0-5_ on *t*_nap-5-10_ and reported Pearson correlation and Cohen’s *f*^2^ as a measure of global effect size [36]. To assess whether distal skin temperature changes that occurred before naps varied by age group, we stratified and plotted the difference between median distal skin temperature during *t*_nap-5-10_ and *t*_nap-0-5_ for older (*>*65; *n* = 1507) and younger individuals (*<*30; *n* = 2131). To assess heat loss prior to nap onset, we compared distal skin temperature during *t*_nap-5-10_ and *t*_nap-0-5_ within these older and younger groups using the Wilcoxon signed-rank test. We also used the Mann–Whitney *U* test and Cliff’s *δ* to compare changes in distal skin temperature that occurred before naps between older and younger individuals.

#### Distal skin temperature changes from before to during naps

To describe changes in distal skin temperature during naps, we repeated the above approaches (*Distal Skin Temperature Changes Before Naps*) using 5-min distal skin temperature segments at *t*_nap-5-10_ (5–10 min prior to nap) and *t*_nap + 10-15_ (10–15 min into nap onset). We also compared median distal skin temperature at *t*_wake+180-240_ (180–240 min after wake time) to median distal skin temperature at *t*_nap + 10-15_ to examine changes from a more stable, morning baseline temperature that may avoid potential impacts of overnight sleep and meals (i.e. breakfast and lunch) on distal skin temperature [37]. We only used days where *t*_wake+180-240_ occurred before *t*_nap + 10-15_.

## Results

### Cohort composition

As described in *Methods*, of the 66 664 participants comprised the initial participant pool in the parent TemPredict study [26], 20 027 met our inclusion criteria (see subsection in *Methods, Data Preparation, and Selection*). This cohort of 20 027 individuals mainly comprised white individuals from North America or Europe (Table 1).

**Table 1:** Demographics of individuals in this study

Sex	*n =* 20 027
Male, *n* (%)	11 758 (59%)
Female, *n* (%)	8256 (41%)
Other, *n* (%)	13 (<1%)
Age (years)	
18–19, *n* (%)	60 (<1%)
20–29, *n* (%)	1671 (8.3%)
30–39, *n* (%)	4891 (24.4%)
40–49, *n* (%)	6028 (30.1%)
50–59, *n* (%)	4508 (22.5%)
≥60, *n* (%)	2869 (14.3%)
Race	
White, *n* (%)	17 298 (86%)
Asian, *n* (%)	957 (4.8%)
Hispanic, *n* (%)	906 (4.5%)
South Asian, *n* (%)	302 (1.5%)
Black, *n* (%)	239 (1.2%)
Middle Eastern, *n* (%)	190 (<1%)
Native American, *n* (%)	97 (<1%)
Native Hawaiian, *n* (%)	59 (<1%)
Other, *n* (%)	752 (3.8%)
Geographical location	
North America, *n* (%)	13 105 (65%)
Europe, *n* (%)	5809 (29%)
Oceania, *n* (%)	553 (2.8%)
Asia, *n* (%)	418 (2.1%)
South America, *n* (%)	64 (<1%)
Africa, *n* (%)	30 (<1%)
Undisclosed, *n* (%)	48 (<1%)

Individuals were allowed to identify with more than one race.

### Descriptive characteristics

Data collection spanned an 11-month period from January 1, 2020 to November 30, 2020, capturing potential seasonal variations. We report counts and durations as median [IQR] and clock times as mean (SD): overall, the median number of days with any data (e.g. sleep windows, distal skin temperature, etc.) recorded was 242 [184, 283] days per individual. Individuals in this cohort tended to wear the Oura Ring regularly, as the median number of consecutive days for which there was any data was 144 [63, 244] days per individual. The median number of overnight sleep windows was comparable to the number of days with any data at 222 [160, 244] nights per individual. The median number of nap windows per individual was 51 [25, 93] days. The mean nap time occurred at 15:03 (158 min). The median duration of nap sleep windows was 0.52 [0.45, 0.62] h. The mean overnight sleep onset time occurred at 23:09 (76 min.). The mean overnight wake onset time occurred at 7:19 (85 min). The median duration of overnight sleep windows was 8.12 [7.67, 8.55] h.

### Daily nap probability

**Figure 2:**
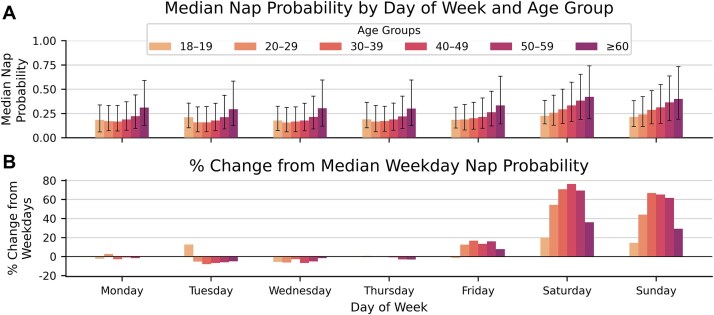
Patterns of napping. (A) Median probability of naps with IQR by age group and day of week. (B) Percent change in median probability of naps compared with the median weekday probability of naps by age group and day of week.

We included 19 909 individuals in this analysis, as they met the criterion of having at least one period of daytime Oura Ring wear per day of week. Age groups varied in numbers of participants for the computation of daily nap probability as follows: 18–19 (*n =* 58), 20–29 (*n =* 1657), 30–39 (*n =* 4846), 40–49 (*n =* 5999), 50–59 (*n =* 4492), and ≥60 years (*n =* 2857). Individuals tended to nap more frequently as age increased, with individuals in the ≥60 years age group napping the most frequently across all days of the week; individuals in the 18–39 years age group tended to nap the least frequently (Figure 2A). Holding day of week constant, a 1-year increase in age corresponded to an increase in daily nap probability of 0.0052 (95% CI [0.0049 to 0.0055]; *p* < .0001). Holding age constant, the probability of naps also increased by 0.116 on weekends compared with weekdays (95% CI [0.114 to 0.118]; *p* < .0001).

As shown in Figure 2B, individuals in the ≥60 years age group had a smaller percent increase in naps during weekends relative to weekdays than all other age groups except for the 18–19 years age group. Individuals in the 30–39 and 40–49 years age group had the largest percent increase in naps from weekdays to weekends, with the percentage tapering downwards from the peak evident within these age groups.

### Daily sleep probability

**Figure 3:**
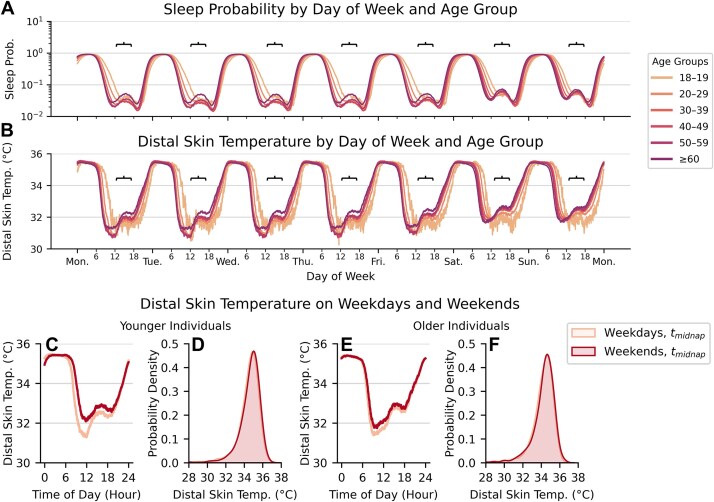
Daily sleep probability and distal skin temperature across the week. (A) Age-stratified daily sleep probability. (B) Age-stratified daily distal skin temperature. Black bars indicate concurrent daytime increases in both sleep probability and distal skin temperature, which tend to coincide. (C) Median 24-h distal skin temperature and (D) distribution of median distal skin temperature during *t*_midnap_ on weekends and weekdays for younger individuals. (E) Median 24-h distal skin temperature and (F) distribution of median distal skin temperature during *t*_midnap_ on weekends and weekdays for older individuals.

We included all 20 027 individuals in these analyses, as they had available sleep windows on each day of the week. We plotted results by age group; the following numbers of participants fell into each group: 18–19 (*n =* 60), 20–29 (*n =* 1671), 30–39 (*n =* 4891), 40–49 (*n =* 6028), 50–59 (*n =* 4508), and ≥60 years of age (*n =* 2869). Figure 3A shows that across all individuals, sleep probability appeared higher on weekends during the daytime compared with weekdays.

### Daily distal skin temperature

We included 14 850 individuals in these analyses who had distal skin temperature during the midpoint of naps (*t*_midnap_) on each day of the week. Holding day of week constant, we found that a 1-year increase in age corresponded to a decrease in distal skin temperature during *t*_midnap_ of −0.0084°C (95% CI [−0.010 to −0.0070]; *p* < .0001). Holding age constant, distal skin temperature at *t*_midnap_ increased by 0.25°C on weekends compared with weekdays (95% CI [0.24 to 0.27]; *p* < .0001). We plotted results by age group; the numbers of participants in each group were identical to those in the analysis of *Daily Sleep Probability* (immediately above). As shown in Figure 3B, the increase in midday distal skin temperature coincided with the increase in sleep probability in Figure 3A. Furthermore, both sleep probability and distal skin temperature rhythms of younger age groups appear phase-delayed compared with older individuals (Figure 3A and B).

We contrasted distal skin temperature on weekends and weekdays for younger (≤30 years) and older individuals (≥65 years): median distal skin temperature during *t*_midnap_ was higher for both younger and older individuals on weekends relative to weekdays (Figures 3C–F; Table 2). There was a larger effect of weekends on distal skin temperature during *t*_midnap_ among younger individuals (*δ =* 0.07) compared with older individuals (*δ =* 0.05). Older individuals had significantly lower distal skin temperature during *t*_midnap_ relative to younger individuals on both weekends (*p* < .0001; *δ =* −0.20) and weekdays (*p* < .0001; *δ =* −0.19).

**Table 2:** Comparison of distal skin temperature during naps (*t*_midnap_) on weekdays versus weekends

	Younger individuals			Older individuals		
Day of distal skin temperature measurements	Median [IQR]	*P*-value	Cliff’s *δ*	Median [IQR]	*P*-value	Cliff’s *δ*
Weekdays	34.71 [34.06, 35.20]	<.0001	0.07	34.40 [33.73, 34.93]	.012	0.05
Weekends	34.83 [34.13, 35.32]			34.49 [33.80, 35.02]		

*t*_midnap_, midpoint time of each nap window.

### Nap length and distal skin temperature

**Figure 4:**
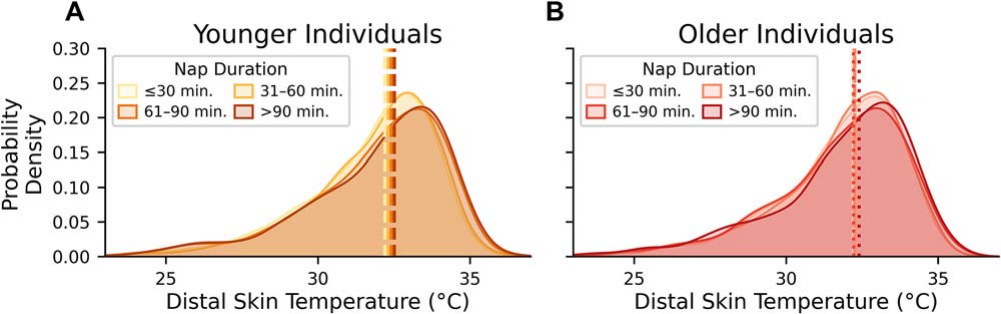
Association between nap length distal skin temperature. (A) Median distal skin temperature 60 min before naps for younger and (B) older individuals.

Among younger individuals (≤30 years), sample sizes by nap duration were: ≤30 min (*n* = 2080), 31–60 min (*n* = 2035), 61–90 min (*n* = 1778), and >90 min (*n* = 1793). For older individuals (≥65 years), sample sizes were: ≤30 min (*n* = 1484), 31–60 min (*n* = 1471), 61–90 min (*n* = 1376), and >90 min (*n* = 1317). We observed that longer naps were generally preceded by higher distal skin temperature before the nap among both younger and older individuals (Figure 4A and B; Table 3). Distal skin temperature was significantly lower during the 60 min preceding the shortest naps (≤30 min) compared with longer naps among younger individuals. In contrast, among older individuals, distal skin temperature prior to naps was significantly higher than the ≤30-min reference only for naps exceeding 90 min in length.

**Table 3:** Comparison of median distal skin temperature before naps of various durations relative to naps of length ≤30 min

	Younger individuals			Older individuals		
Nap length	Distal skin temp (°C)	*P*-value	Cliff’s *δ*	Distal skin temp. (°C)	*P*-value	Cliff’s *δ*
≤30 min	32.20	–	–	32.21	–	–
31–60 min	32.32	.045	0.04	32.25	.45	0.02
61–90 min	32.41	.00020	0.07	32.22	.61	0.01
>90 min	32.51	<.0001	0.09	32.40	.022	0.05

### Distal skin temperature changes before and during naps

#### Before naps

**Figure 5:**
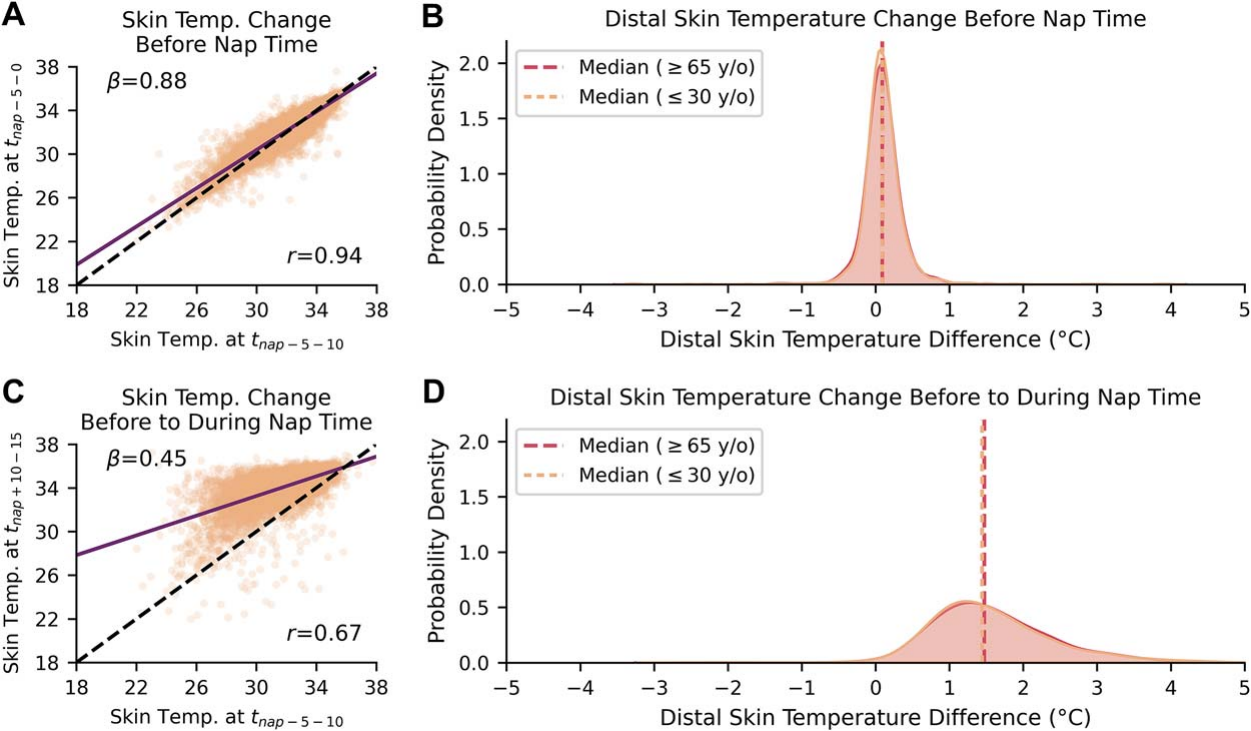
Changes in distal skin temperature before/during naps. (A) Comparison of distal skin temperature before a nap (*t*_nap-0-5_ vs. *t*_nap-5-10_). Regression line (purple), regression coefficient (*β*), and Pearson correlation (*r*) for the depicted data are shown. (B) Distribution of differences in median distal skin temperature between *t*_nap-0-5_ and *t*_nap-10-15_. (C) Comparison of distal skin temperature before (*t*_nap-5-10_) to during (*t*_nap + 10-15_) naps. (D) Distribution of differences in median distal skin temperature between *t*_nap + 10-15_ and *t*_nap-5-10_.

Across all individuals, median distal skin temperature 5 to 10 min before nap onset time (*t*_nap-5-10_) was closely related to distal skin temperature 0 to 5 min before nap onset time (*t*_nap-0-5_; Table 4; Figure 5A). Notably, median distal skin temperature during *t*_nap-0-5_ was significantly higher than distal skin temperature during *t*_nap-5-10_ (*p* < .0001) for both older and younger individuals. However, the effect size was very small (*δ* = 0.02 for both older and younger individuals). The difference between distal skin temperature during *t*_nap-5-10_ and *t*_nap-0-5_ was not significantly different for older (≥65 years) versus younger individuals (≤30 years; Table 5; Figure 5B).

**Table 4:** Association between distal skin temperatures before and during naps

Regression analysis condition	*β* [95% CI]	*P*-value	*r*	*f* ^2^
Before naps	0.876 [0.871 to 0.880]	<.0001	0.94	7.58
Before to during naps	0.451 [0.444 to 0.458]	<.0001	0.67	0.80

**Table 5:** Association between distal skin temperatures before and during naps stratified by older and younger individuals

	Younger individuals	Older individuals		
Condition	Median [IQR]	Median [IQR]	*P*-value	Cliff’s *δ*
Before naps	0.08 [−0.03, 0.21]	0.09 [−0.03, 0.21]	.91	−0.002
Before to during naps	1.44 [1.01, 2.02]	1.47 [1.03, 2.05]	.47	0.01

#### Before naps to during naps

Across all individuals, median distal skin temperature during *t*_nap-5-10_ was less closely matched with distal skin temperature 10 to 15 min after nap onset time (*t*_nap + 10-15_; Table 4; Figure 5C). The difference between distal skin temperature during *t*_nap-5-10_ and *t*_nap + 10-15_ was not significantly different for older versus younger individuals (Table 5; Figure 5D). We observed a similar association between distal skin temperature during *t*_nap-5-10_ with distal skin temperature 180 to 240 min after awakening from overnight sleep (*t*_wake+180-240_; Supplementary Figure S1; Supplementary Table S2).

## Discussion

The present study used high-resolution, real-world wearable data among a large population composed of different age groups to identify patterns of napping behavior and associated physiological changes. Older age groups (≥60 years) consistently napped more frequently across the week, whereas younger age groups (30–49 years) showed the greatest increase in napping on weekends. Distal skin temperature rose similarly from just before nap onset to early into the nap (i.e. from 5–10 min before napping to 10–15 min after nap onset time) for both younger and older individuals; however, absolute distal skin temperature during the midpoint of naps was higher among younger individuals than older individuals. Greater distal skin temperature typically preceded longer naps, further supporting an association between distal skin temperature and sleep pressure. Across all naps, median distal skin temperature 0–5 min before nap onset was slightly higher than 5–10 min before nap onset, potentially indicating a mild increase in heat dissipation from the extremities prior to nap onset. Taken together, our findings highlight the potential for distal skin temperature around and during naps to serve as a passive biomarker of sleep pressure and age-related changes in physiology.

Our observation that greater increases in distal skin temperature often preceded longer naps among both younger and older individuals suggests that increases in distal skin temperature before naps may scale with sleep pressure, potentially serving as a passive indicator of the body’s desire or inclination for sleep. Given that individuals may not be able to nap as long as they please, the distal skin temperature we observed before shorter naps (≤30 min) may reflect higher underlying sleep pressure than a short nap would suggest, particularly if these naps were truncated due to time constraints. Our data, however, cannot address this hypothesis. Thus, our findings support the use of well-timed, shorter naps to compensate for sleep debt [10]. These findings underscore the potential for wearable-assessed distal skin temperature to provide passive insight into fluctuations in sleep pressure and daytime sleep–wake regulation.

One might expect distal skin temperature prior to naps to correlate more strongly with nap duration in older individuals who tend to nap more frequently, given the prior data showing an association between elevated distal skin temperature and greater subjective sleepiness [38] and shorter sleep onset latency [39]. Instead, we observed that younger individuals had higher distal skin temperature in the hour before longer naps, whereas older individuals showed smaller differences in distal skin temperature in the hour before naps of different durations. These smaller differences among older individuals may reflect age-related changes in thermoregulatory range, such as reduced vasoconstrictive capacity [28]. Although healthy older adults typically do not experience greater daytime sleepiness than younger adults, daytime sleepiness may become more prevalent with age due to an increased burden of health conditions, which can disrupt nighttime sleep and increase tendencies toward daytime napping [40]. Future studies should disentangle the relative contributions of sleep pressure and age-related changes in thermoregulatory range to these distal skin temperature patterns before naps. Our findings suggest that elevated daytime distal skin temperature—whether reflecting sleep pressure, impaired thermoregulation, or both—may serve as a passive biomarker suggestive of physiological disruption [41]. Although future studies should validate our findings using self-reported napping information as well as labeled clinical data, these findings underscore the potential of wearable-derived distal skin temperature metrics to inform early detection of age-related conditions such as ADRD, which is associated with both increased daytime sleepiness [42] and both physiological and behavioral disruptions [43].

If greater increases in distal skin temperature reflects greater sleep pressure, limiting the opportunity to nap may have important health consequences. Suppressing naps under such conditions may be harmful, particularly when individuals are compensating for accrued sleep debt [44] or when daytime sleepiness reflects underlying health conditions [45]. Naps have been shown to improve heart rate variability, an indicator of cardiovascular disease risk [46], suggesting that naps may confer cardiovascular benefits. This may be especially relevant for older individuals, who face both greater disease risk and already exhibit a tendency to nap; however, frequent or prolonged napping may also indicate underlying health risks [12, 13]. Prescriptive napping for improved health and disease risk mitigation remains a critical area for future research.

The findings in this study are subject to several limitations. While the Oura Ring is able to detect sleep windows throughout the day, it is not primarily a nap-detection device [47]. A notable example of this limitation is the steep increase in detected nap windows beginning around 18:30, peaking near 21:40, and declining toward midnight, which likely reflects misclassification near habitual bedtime rather than true increases in napping behavior. For this reason, we included data preprocessing steps to exclude short sleep windows that may be due to inactivity, exclude sleep windows occurring near habitual bedtimes when misclassification of naps may be most likely, and merge nearby sleep windows that may be part of longer, continuous sleep windows. Although device-related errors in detection of nap windows are possible, we do not expect these errors to vary systematically by age group. Given the clear, near-monotonic increase in daily nap frequency with age, we are confident that the overall direction of the observed association is robust.

Anthropometric, physiological, and hormonal differences between male and female individuals may influence distal skin temperature under different conditions. High ambient temperatures associated with transient heat exposure [48], potentially due to warmer seasons, can elevate distal skin temperature. Evidence for overall sex differences in distal skin temperatures is mixed [49–51], but distal temperature varies across the female lifespan: distal skin temperatures is generally higher during the luteal (vs. follicular) phase of the menstrual cycle during sleep [52, 53], alters with contraceptive use [54], elevates and loses rhythmicity during pregnancy [55], and increases during hot flashes in peri- and post-menopause [56]. Thus, sex-related hormonal factors contribute to variation in distal skin temperature [57, 58]. Female individuals typically have a higher surface area-to-mass ratio [59] but also higher body fat percentages that insulate against heat transfer from the core to peripheral regions [60], with possible compensatory heat loss through the extremities [61]. Greater resting vasoconstrictive capacity [62], delayed sweating [63], and lower evaporative cooling rates [63] may also contribute to higher skin temperatures in females [48]. Thus, sex-related differences should be considered as potential sources of variability in distal skin temperature measurements and accounted for when possible.

Several common afternoon activities may also influence distal skin temperature. Food intake around lunchtime may contribute to distal skin temperature rises during the daytime [64]. While sleep initiation is associated with increases in distal skin temperature [65], simply lying down can also redistribute heat from the core to the periphery [66], so postural recumbency during rest or attempted naps may elevate distal skin temperature even without sleep. Bedding, which frequently accompanies attempts to initiate sleep, insulates against heat loss and creates a warm microclimate between the skin and bedding [67]. Clothing can have a similar effect [68]. Although the Oura Ring is less likely to be covered by clothing than a smartwatch, this remains a relevant consideration. Such routine activities may introduce variability that is independent of underlying physiological processes.

Finally, while we have labels for some age-related diseases (e.g. hypertension), we lack labels for others (e.g. ADRD), and over half of our cohort lacks disease annotations altogether. Therefore, the associations we observe are only suggestive of a role for distal skin temperature and napping patterns in early disease screening. Importantly, distal skin temperature and napping patterns were highly heterogenous across individuals. Detection of individual-level changes in either of these metrics may help identify those at greater risk for age-related disease, supporting targeted care. Many follow-up investigations are required before distal skin temperature or napping metrics from wearable devices might be used for early risk assessment. Nonetheless, our findings demonstrate the feasibility of large-scale nap-related investigations using wearable sensors in real-world settings.

## Supplementary Material

Soltani_et_al_Supplementary_Material-_2026-02-19_zsag077

## Data Availability

Oura’s data use policy does not permit us to make wearable device data (collected via the Oura Ring) available to third parties. Access to anonymized and privacy-protected data may be granted to a qualified academic investigator upon completing agreements with Oura Health Oy and the investigators. Please contact Ashley E. Mason and Benjamin L. Smarr to obtain an application to obtain these data.
