# Supplementary material for: Age-related patterns in distal skin temperature during naps
Source: Sleep. 2026 Mar 17;49(6):zsag077. doi: 10.1093/sleep/zsag077 (PMC13267894; doi:10.1093/sleep/zsag077)
Supplement: Soltani_et_al_Supplementary_Material-_2026-02-19_zsag077 [file soltani_et_al_supplementary_material-_2026-02-19_zsag077.docx]

Age-Related Patterns in Distal Skin Temperature During Naps

Severine Soltani^1,2^, Wendy Hartogensis^3^, Patrick Kasl^2^, Subhasis Dasgupta^4^, Stephan Dilchert^5^, Frederick M. Hecht^3^, Benjamin L. Smarr^2,6,†^, Ashley E. Mason^3,†,*^

^1^Bioinformatics and Systems Biology Graduate Program, University of California San Diego, La Jolla, USA

^2^Shu Chien‑Gene Lay Department of Bioengineering, University of California San Diego, La Jolla, USA

^3^Osher Center for Integrative Health, University of California San Francisco, San Francisco, USA

^4^San Diego Supercomputer Center, University of California San Diego, La Jolla, USA

^5^Zicklin School of Business, Baruch College, The City University of New York, New

York, USA

^6^Halıcıoğlu Data Science Institute, University of California San Diego, La Jolla, USA

^†^These authors contributed equally to this work.

^*^Corresponding author: Ashley E. Mason, PhD. Osher Center for Integrative Health, University of California, San Francisco, San Francisco, 1545 Divisadero St, 4^th^ floor, CA 94115, USA. Email: Ashley.Mason@ucsf.edu.

Table of Contents

[Definitions, Data Preparation, and Data Selection 3](#_Toc215607580)

[Table S1. Python Packages, Versions, and Their Applications in This Study. 8](#_Toc215607581)

[Figure S1. Changes in distal skin temperature before to during naps. 9](#_Toc215607582)

[Table S2. Association Between Distal Skin Temperatures Before (*t*_wake+180-240_) and During (*t*_nap+10-15_) Naps. 10](#_Toc215607583)

[References 11](#_Toc215607584)

# Definitions, Data Preparation, and Data Selection

**Definitions**

***Overnight sleep window***

We defined an overnight sleep window as any period of sleep within a 24-hour period that:

1. Is the longest duration of sleep within a 24-hour period, as labeled by the Oura Ring. If other nearby sleep windows were merged with this overnight sleep window (see subsection *Data Preprocessing* under section Data Preparation), the final merged sleep window is considered the overnight sleep window.
   1. Importantly, if there was no longest sleep window identified by the Oura Ring for a given 24-hour period, we did not promote the longest short sleep window to overnight sleep.

***Nap window***

We defined a nap window as any period of sleep within a 24-hour period that:

1. Is shorter than the overnight sleep window.
2. Is at least 15 minutes in duration.
3. Is allowed to occur more than once per 24-hour period.
4. Does not occur within 4 hours of the same day’s bedtime.

**Data Preparation**

The initial participant pool from Mason *et al.* (2024) included 66,664 individuals [1]. We used the same filtering procedures to arrive at this baseline cohort of 66,664 individuals (see Figure 1 in main text).

***Initial Data Availability***

1. **Sleep window data**: For all subsequent data preparation steps, we included only individuals with sleep window data available (*n*=46,354). Of the *n*=20,310 filtered individuals,
   1. *n*=20,289 lacked all Oura Ring data (*i.e.*, no minute-level data or overnight “sleep summary” data). This potentially represents individuals who opted out of the study after the initial baseline demographics assessment or later opted out of sharing their data.
   2. *n*=4,330 were not able to have their Oura ID matched to their study ID, rendering their Oura data inaccessible for this study.

***Sleep Window Data Format Provided by Oura***

Oura provided 14,267,046 sleep window entries for 46,354 individuals with start and end timestamps and a binary label that identified the longest sleep window (*i.e.*, typically overnight sleep) within a 24-hour window; all other sleep windows detected within that 24-hour period are retained but not labeled as the longest sleep window (*i.e.*, they are effectively nap windows). Below, we take several steps to further preprocess the Oura-provided sleep entries to better align with our definitions of “overnight sleep” and “nap” windows described in **Definitions**.

***Data Preprocessing***

1. **Merging nearby sleep windows**: First, we merged sleep windows within 15 minutes of each other in the event that there were duplicated sleep windows or brief periods of wakefulness, so that, for example, fragmented sleep would not result in naps being identified in the middle of a disrupted nighttime sleep window. In practice, this means that two successive sleep windows separated by, for example, 10 minutes would be merged into a single sleep window. This step was intended to bridge short gaps in sleep that may have been inappropriately split by the Oura Ring into two separate sleep windows. Such instances may include a brief awakening to use the restroom during overnight sleep or a nap on the couch before transitioning to bed for overnight sleep. This would merge these disparate windows into one continuous sleep window rather than a nap and overnight sleep (or two naps). This merging reduced our sleep window count by 1,351,063 windows.
2. **Filtering out short sleep windows**: After all merges, we filtered out sleep windows of durations shorter than 15 minutes to mitigate against the possibility of the Oura Ring designating periods of inactivity as sleep. This step filtered out 2,256,823 sleep windows and 1 individual for whom this step eliminated all sleep windows.
3. **Relabeling sleep windows**: Following duration thresholding, we re-identified the longest sleep window within a 24-hour period. The longest sleep window in a given 24-hour period was designated the overnight sleep window (*i.e.*, the longest sleep window). Importantly, to prevent naps being identified as overnight sleep windows, sleep windows composed of only naps (*e.g.*, a single nap or two merged naps) would not be labeled as the overnight sleep window even if they are the only sleep windows in a 24-hour period. This is intended to mitigate against erroneously labeling naps as overnight sleep during instances in which individuals lack overnight sleep windows but have daytime sleep windows (*e.g.*, did not wear the Oura Ring during overnight sleep but did wear it during the day).
4. **Filtering out sleep windows too close to overnight sleep**: We iterated over each individual’s bedtime windows and filtered out naps that ended within 4 hours of bedtime onset time to ensure that sleep windows possibly representing polyphasic sleep patterns were not labeled as naps. Our intention was to focus on the thermoregulatory aspects surrounding naps (which often—but not necessarily— occur during the afternoon) rather than shorter evening sleep windows that may reflect polyphasic nighttime sleep patterns or the start of a longer overnight sleep window. In addition, we observed a sizeable proportion of nap windows beginning shortly before (~18:30, peaking at ~21:40) typical bedtimes, which may reflect false positives due to the Oura sleep staging algorithm being primed to detect sleep towards the evening, potentially resulting in some erroneous sleep windows. To take a conservative approach, we filtered out these sleep windows close to bedtime. If no overnight sleep window was recorded for a given day, we computed the individual’s mean bedtime onset time separately for weekdays and weekends and used the appropriate mean bedtime onset time in place of the missing overnight sleep window. This step filtered out 1,833,656 sleep windows.

**Data Selection**

After preprocessing sleep window data, we applied several exclusion criteria to arrive at our final analytic cohort:

1. Filtered out individuals who did not report age information (*n*=1,042).
2. Filtered out individuals who self-identified shift workers (*n*=484).
3. Filtered out individuals who reported being pregnant (*n*=222).
4. Filtered out individuals who only have data available on days where symptoms of illness or active diagnoses were reported (*n*=4).
5. Filtered out individuals with fewer than 100 24-hour periods in which at least 8 hours of distal skin temperature data were recorded (*n*=23,156).
6. Filtered out individuals without any nap windows (*n*=1,418).

Our final analytic cohort was composed of the remaining 20,027 individuals.

| Table S1. Python Packages, Versions, and Their Applications in This Study. | | |
| --- | --- | --- |
|  |  |  |
| Package | Version | Application |
| cliffs_delta | 1.0.0 | Effect size measure |
| multiprocessing | — | Parallel computing |
| numpy [2] | 2.0.2 | Data structures; descriptive statistics |
| pandarallel | 1.6.5 | Parallel computing |
| pandas [3,4] | 2.2.3 | Data structures; descriptive statistics |
| pycountry-convert | 0.7.2 | Geographical location |
| scipy [5] | 1.15.1 | Statistical hypothesis testing; correlations; low-pass filtering; peak-finding |
| statsmodels [6] | 0.14.4 | Linear and linear mixed model regressions |


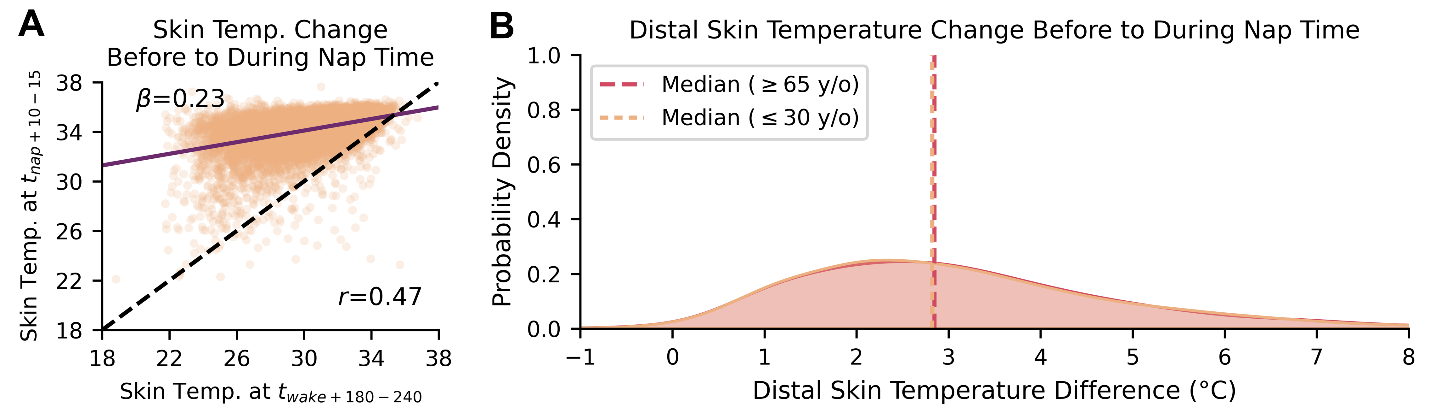


# Figure S1. Changes in distal skin temperature before to during naps.

(A) Comparison of distal skin temperature before a nap (*t*_nap+10-15_ vs. *t*_wake+180-240_). Regression line (purple), regression coefficient (𝛽), and Pearson correlation (*r*) for the depicted data are shown. (B) Distribution of differences in median distal skin temperature between *t*_nap+10-15_ vs. *t*_wake+180-240_.

| Table S2. Association Between Distal Skin Temperatures Before (*t*_wake+180-240_) and During (*t*_nap+10-15_) Naps. | | | | | |
| --- | --- | --- | --- | --- | --- |
| Regression analysis | | | | | |
| β [95% CI] | | *P*-value | | *r* | *f*^2^ |
| 0.234 [0.228, 0.240] | | <.0001 | | 0.47 | 0.28 |
|  | | | | | |
| Age-stratified differences | | | | | |
| Younger individuals  Median [IQR] |  | Older individuals  Median [IQR] |  | *P*-value | Cliff’s *δ* |
|  |  |  |  |  |  |
| 2.82 [1.84, 4.07] |  | 2.85 [1.84, 4.07] |  | .79 | 0.01 |

# References

1. Mason AE, Kasl P, Soltani S, et al. Elevated Body Temperature Is Associated with Depressive Symptoms: Results from the Tempredict Study. *Sci Rep*. 2024;14(1):1884. doi:10.1038/s41598-024-51567-w

2. Harris CR, Millman KJ, van der Walt SJ, et al. Array Programming with NumPy. *Nature*. 2020;585(7825):357-362. doi:10.1038/s41586-020-2649-2

3. Reback J, McKinney W, Van Den Bossche J, et al. pandas-dev/pandas: Pandas 1.0. 5. *Zenodo*. Published online 2020.

4. McKinney W. Data structures for statistical computing in Python. In: Vol 445. 2010:51-56.

5. Virtanen P, Gommers R, Oliphant TE, et al. SciPy 1.0: Fundamental Algorithms for Scientific Computing in Python. *Nat Methods*. 2020;17(3):261-272. doi:10.1038/s41592-019-0686-2

6. Seabold S, Perktold J. Statsmodels: Econometric and Statistical Modeling with Python. *SciPy*. 2010;7(1).
